# Tissue-specific role and associated downstream signaling pathways of adiponectin

**DOI:** 10.1186/s13578-021-00587-4

**Published:** 2021-04-26

**Authors:** Bipradas Roy, Suresh Selvaraj Palaniyandi

**Affiliations:** 1 Division of Hypertension and Vascular Research, Department of Internal Medicine, Henry Ford Health System, Integrative Biosciences Center (IBio), Room #3402, 6135 Woodward, Detroit, MI 48202 USA; 2 Department of Physiology, Wayne State University, Detroit, MI 48202 USA

**Keywords:** Metabolic syndrome, Adiponectin, AMPK, Vascular endothelial cell, Cardiomyocyte, VSMC

## Abstract

According to the World Health Organization, metabolic syndrome (MetS) can be defined as a pathological condition characterized by abdominal obesity, insulin resistance, hypertension, and hyperlipidemia. The incidence of MetS keeps rising, as at least 35% of the USA population suffers from MetS. One of the worst comorbidities of metabolic syndrome are cardiovascular diseases that significantly amplifies the mortality associated with this syndrome. There is an urgent need to understand the pathophysiology of MetS to find novel diagnosis, treatment and management to mitigate the MetS and associated complications. Altered circulatory adiponectin levels have been implicated in MetS. Adiponectin has numerous biologic functions including antioxidative, anti-nitrative, anti-inflammatory, and cardioprotective effects. Being a pleiotropic hormone of multiple tissues, tissue-specific key signaling pathways of adiponectin will help finding specific target/s to blunt the pathophysiology of metabolic syndrome and associated disorders. The purpose of this review is to elucidate tissue-specific signaling pathways of adiponectin and possibly identify potential therapeutic targets for MetS as well as to evaluate the potential of adiponectin as a biomarker/therapeutic option in MetS.

## Introduction

Metabolic diseases including type 2 diabetes, obesity and other cardiovascular diseases are becoming alarming throughout the entire world among people of all age groups (~ 25% of all adults in the world) and causing millions of deaths each year [1]. In accordance with the prevalence of metabolic diseases like diabetes, cardiovascular diseases (CVDs) and other comorbidities are increasing day-by-day as well (relative risk of CVD is twofold in men and threefold in women) [2]. Thus, finding a common regulatory factor/mechanism which governs both metabolic conditions and associated CVDs will provide new therapeutic options for managing them effectively. Adiponectin, a 244 amino acid adipocytokines, qualifies this criterion. Adiponectin is encoded by the ADIPOQ gene in humans and is predominantly secreted by the adipocytes of brown adipose tissue. There are a wide range of cells and tissues such as osteoblasts, parenchymal cells, myocytes, epithelial cells, hepatocytes, and placental tissue that secret adiponectin in a small amount [3].

Four different forms of adiponectin have been identified in human plasma so far including low-molecular-weight (LMW) trimer, medium-molecular-weight (MMW) hexamer, high-molecular-weight (HMW) oligomer and globular adiponectin (gAd) [4, 5]. Post translational modifications in adiponectin account for more than eight isoforms of adiponectin [6]. The molecular weight of LMW, MMW and HMW adiponectin are 67 kDa, 140 kDa and 300 kDa respectively. LMW adiponectin has a half-life of 32 min while MMW and HMW adiponectin have a half-life of 83 min. The plasma concentration of adiponectin in humans ranges from 2 to 20 µg/ml, which is around 1000-fold higher than the plasma concentration of insulin [4]. A vast majority of studies suggest that HMW adiponectin is the predominant form contributing to the regulation of different types of biological functions including insulin sensitivity, glucose uptake, and lipid metabolism [7]. Due to tissue specific variability in the expression of adiponectin receptors and diversity of adiponectin isoforms, the tissue specific adiponectin signaling mechanism also varies. In this review, we provide potential cellular mechanisms through which adiponectin exerts its role in a wide range of cell types.

## Pleiotropic role of adiponectin

### Adiponectin aided regulation of skeletal muscle function

Skeletal muscle contributes to almost half of the human body mass and is primarily known for its role in physical movement, posture, and breathing. However, skeletal muscle plays an active role in numerous metabolic pathways including carbohydrate, protein, and fat metabolism and storage of glucose and proteins [8]. Loss of skeletal muscle is associated with delayed recovery from illness and wound healing, decreased resting metabolic rate, higher chance of physical disability and higher health care costs [8, 9]. Findings from several studies showed that alterations of skeletal muscle mass (SMM) are associated with cardiovascular health and even different muscle morphology [9, 10]. A recent finding from a population-based study demonstrated that skeletal muscle mass is inversely proportional to the incidence of CVD [9].

**Fig. 1:**
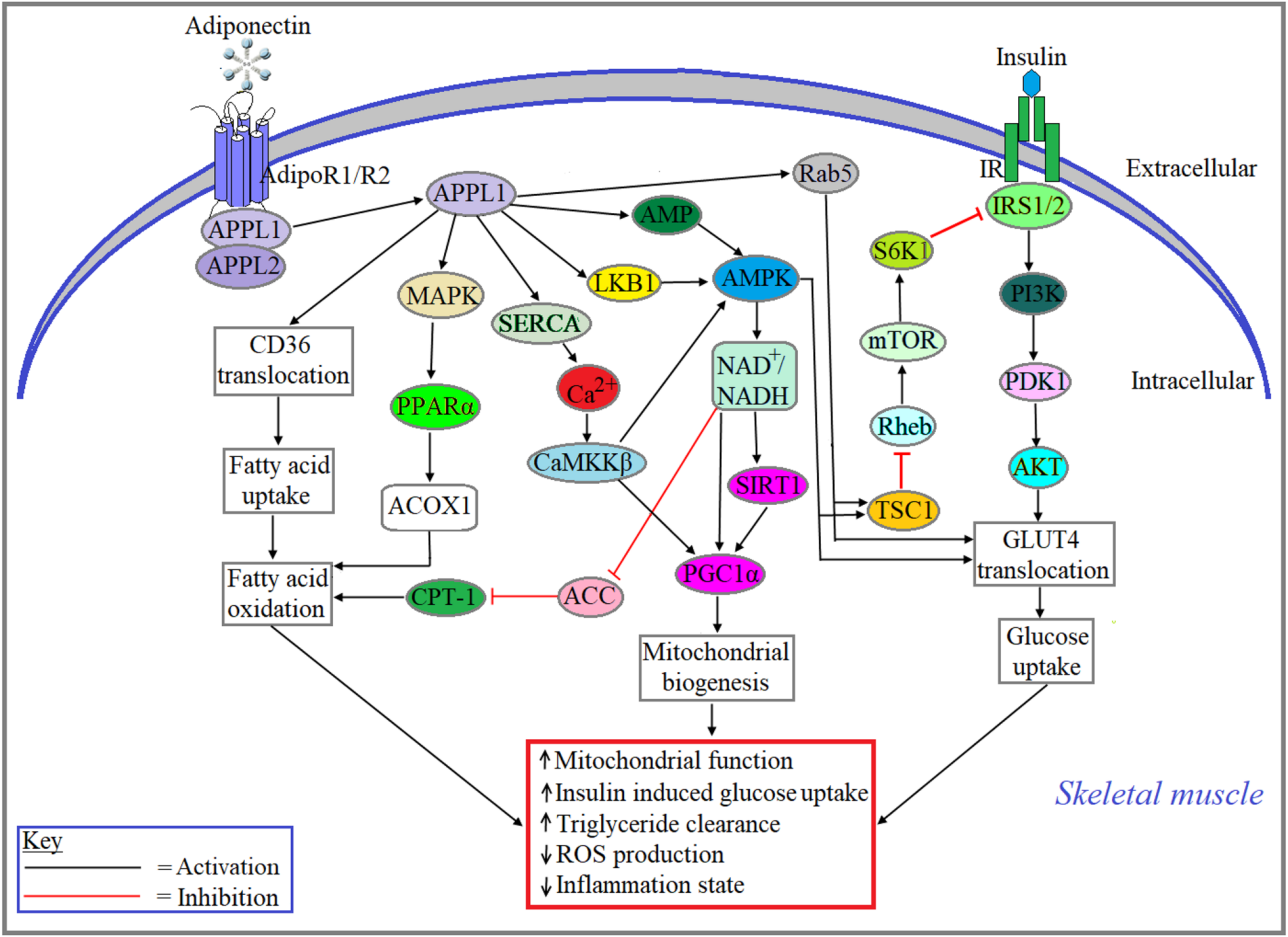
Role of adiponectin in skeletal muscle. Adiponectin-induced activation of AdipoR1/AdipoR2 and subsequent interaction with adapter proteins, APPL1/APPL2, enhance fatty acid oxidation through MAPK-PPARα-mediated expression and activation of ACOX1 and AMPK-NAD^+^/NADH-mediated response of ACC. Activated AdipoR1/AdipoR2 also increase mitochondrial biogenesis through APPL1-SERCA-Ca^2+^-CaMKKβ or LKB1-AMPK-NAD^+^/NADH-SIRT1-mediated activation of PGC1α. Additionally, activation of adiponectin receptors enhances glucose uptake via APPL1-Rab5 or APPL1-AMP-AMPK-mediated translocation of GLUT4 as well as attenuation of Rheb-mTOR-S6K1-induced inhibition of insulin signaling

According to the earlier understanding, adiponectin acts on skeletal muscle cells, secreted only from the adipose tissue as an endocrine manner. However, it has been recently discovered that adiponectin is also produced by the skeletal muscle cells, acting in an autocrine/paracrine manner through the stimulation of adiponectin receptor (AdipoR) 1 and AdipoR2 [11, 12]. Adiponectin plays protective roles in most of the tissue types. However, several studies implied that adiponectin plays both protective and destructive roles in different skeletal muscle types. Adiponectin elevated intracellular Ca^2+^ levels in cultured C2C12 myocytes, which has been reversed by siRNA-mediated knockdown of AdipoR1 [13], thus implying calcium-mediated contractility in muscle cells is regulated by adiponectin. Adiponectin-induced intracellular Ca^2+^ increases mitochondrial biogenesis in skeletal muscle via Ca^2+^/*calmodulin-dependent kinase (CaMK) activation followed by the activation of peroxisome proliferator-activated receptor gamma coactivator 1-alpha* (*PGC1α*) (Fig. 1) [14]. A study by Yoon et al., showed that adiponectin treated differentiated C2C12 cells dose dependently increased the mRNA expression of Acyl-CoA oxidase (ACO) and carnitine palmitoyltransferase 1 (CPT1), two important genes for fatty acid oxidation in skeletal muscle, through the activation of peroxisome proliferator-activated receptor α (PPARα). This study also confirmed that adiponectin-induced activation of PPARα in C2C12 cells is mediated via the activation of AMP-activated protein kinase (AMPK) and p38 mitogen-activated protein kinase (MAPK) which was confirmed by suppression of PPARα activity when C2C12 cells were co-treated with adiponectin and AMPK/p38 MAPK inhibitors. Additionally, adiponectin is shown to inhibit the activity of acetyl-CoA carboxylase (ACC), an endoplasmic reticular enzyme in eukaryotes that can inhibit mitochondrial β-oxidation, through AMPK-mediated pathway in C2C12 cells. Findings from this study suggest that adiponectin increases fatty acid oxidation in skeletal muscle cells through the activation of AMPK/MAPK/PPARα signaling pathway (Fig. 1) [15].

Tibialis anterior muscle (TAM)-specific overexpression of both AdipoR1 and AdipoR2 increased phosphorylation of AMPK, protein kinase B (PKB/AKT) and extracellular signal-regulated kinase (ERK) and subsequently increased the expression of the insulin responsive glucose transporter (GLUT)4 (Fig. 1). However, overexpression of VEGFR2 alone in TAM, increased the expression of PPARα and peroxisomal acyl-coenzyme A oxidase (ACOX)1, the first enzyme of the fatty acid β-oxidation (Fig. 1) [16]. Successive intravenous injections of AdipoRon, an agonist for AdipoR1 and AdipoR2, in C57BL/6J mice decreased the weight gain and mean muscle fiber cross-sectional area and the protein content in plantaris muscle (PLA), but not in soleus muscle (SOL). Additionally, knockdown of AdipoR1 and/or AdipoR2 in C2C12 myotubes rescued AdipoRon-associated decrease in protein content in PLA, but not in SOL. However, AdipoRon administration significantly increased the phosphorylation of AMPK in both PLA and SOL [17]. By employing the adiponectin knockout (AdipoKO) mice, it has been demonstrated that adiponectin is involved in muscle activity as these Adipo KO mice exhibited poor endurance exercise capacity compared with their age-matched wild-type controls [6, 18]. High-fat diet (HFD)-induced diabetes mellitus in rodents impaired gastrocnemius muscle contractile parameters (e.g. peak twitch tension, peak tetanic tension and half relaxation time) by downregulating the gene expression of adiponectin and sarcoplasmic reticulum calcium ATPase (SERCA), a crucial factor of intracellular Ca^2+^ homeostasis. However, adiponectin treatment in diabetic rats increased the expression of SERCA, GLUT4 and adiponectin genes whereas it decreased the levels of serum glucose, insulin, HOMA index, triglycerides, and cholesterol [19]. Also, adiponectin is shown to be associated with increased muscle regeneration and decreased proteolysis which ultimately led to increased muscle mass [20]. Sarcopenia, a pathological condition of progressive muscle loss in aging [21] or cancer [22], is negatively correlated with plasma adiponectin levels [23]. Serum adiponectin levels were significantly lower in a cohort of sarcopenic patients compared with their non-sarcopenic controls [24]. There are contradictory findings to this notion; for instance, elevated adiponectin levels are positively correlated with sarcopenic males with cardiovascular disease (CVD) relative to non-sarcopenic males with CVD [25]. Plasma adiponectin levels were increased significantly in spinal and bulbar muscular atrophy patients compared to the healthy age-matched subjects [26]. Adiponectin is also associated with increased translocation of fatty acids in skeletal muscle cells to fuel β-oxidation process via increased expression of CD36, a fatty acid transporter (Fig. 1) [4]. A significant decrease in mitochondrial biogenesis and function in gastrocnemius muscles by decreasing the expression of PPARα and PGC-1α [27] was observed in AdipoKO mice. The same study also showed that both type 1 (oxidative) and type 2 (glycolytic) muscle fibers are reduced in the skeletal muscle tissues of adipoKO mice. Also, treatment with globular adiponectin decreased ROS formation in skeletal muscle cells [27]. Interactions between phosphotyrosine and an adaptor protein, with PH domain and leucine zipper (APPL)1, which is a downstream signaling protein in adiponectin receptors-mediated signal transduction pathways, play an important role in insulin-mediated signaling in skeletal muscle by activating insulin receptor substrate (IRS) 1/2 [28].

Sirtuin1 (SIRT1) plays an important role in fatty acid oxidation and mitochondrial biogenesis through PGC-1α acetylation and PPAR-α activation in skeletal muscle [29]. It is also shown that adiponectin-induced increase in intracellular Ca^2+^ influx and activation of CaMKK, AMPK, and SIRT1 were decreased in muscle-specific AdipoR1 KO mice [30]. In skeletal muscle, adiponectin-induced activation of tuberous sclerosis 1 (TSC1) inhibits the activity of Ras homolog enriched in brain (Rheb), a negative regulator of insulin signaling as it suppresses the activity of insulin receptor substrate-*1* (IRS-1) (Fig. 1) [31, 32]. Thus, it can be summarized that adiponectin elicits its physiological and ameliorative function in skeletal muscles by modulating aforementioned signaling pathways.

### Adiponectin aided regulation of vascular smooth muscle function

**Fig. 2:**
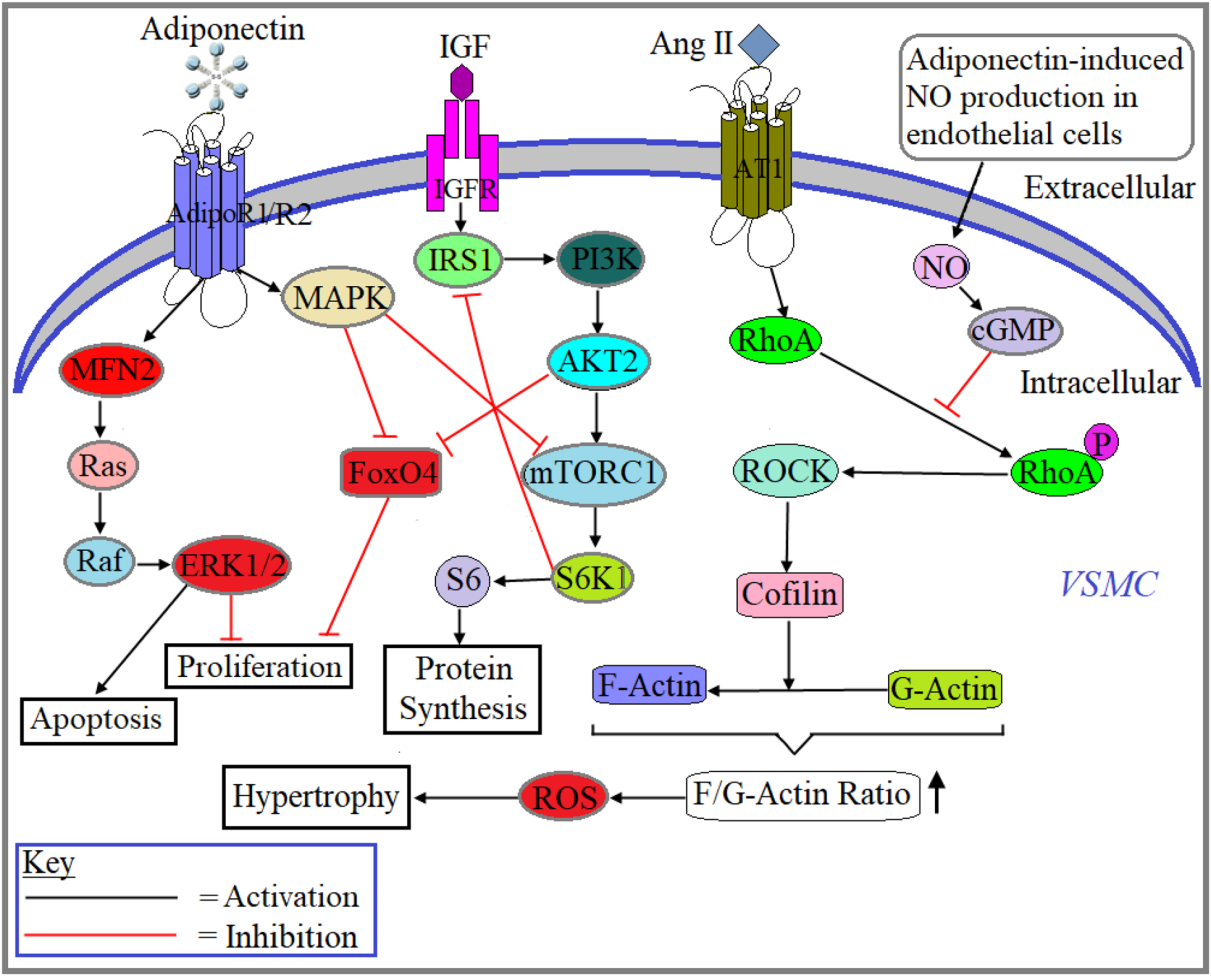
Role of adiponectin in VSMC. Adiponectin enhances apoptosis and inhibits the proliferation of VSMCs through AdipoR1/dipoR2-mediated activation of MFN2-Ras-Raf-Erk1/2 signaling pathway in pathological condition. Adiponectin-induced activation of AMPK attenuates mTORC1-S6K1-mediated inhibition of insulin signaling and subsequently increases protein synthesis and attenuates FoxO4-mediated inhibition of VSMC proliferation in atherosclerosis. Adiponectin-induced production of endothelial NO also diffuses into the VSMCs and subsequently inhibits the activation of RhoA protein, an upstream factor that induces VSMC hypertrophy through ROCK-Cofilin-mediated increase in F-actin/G-actin ratios following the formation of ROS

Vascular smooth muscle cells (VSMCs), key cells in the vessel walls that regulate blood pressure. Their dysfunction may lead to pathological conditions including hypertension, restenosis and atherosclerosis [33]. Adiponectin is an anti-atherogenic and anti-inflammatory adipokine in atherosclerosis [34]. However, the mechanisms underlying the effect of adiponectin on VSMCs are not clearly elucidated. AdipoR1 and AdipoR2 are shown to be expressed in cultured human VSMC line [35]. According to Ding et al., treatment with HMW adiponectin is shown to induce human coronary artery VSMCs differentiation as dictated by the upregulation of contractile protein markers of VSMC differentiation, including smooth muscle myosin heavy chain, h-caldesmon, calponin, and smooth muscle-α-actin [36]. They also showed that HMW adiponectin treatment repressed mechanistic target of rapamycin complex 1 (mTORC1) and forkhead box protein O4 (FoxO4) through the activation of AMPKα2 in human coronary artery VSMCs (Fig. 2) [36]. VSMCs also secret adiponectin to regulate VSMC contractile phenotype in a autocrine/paracrine manner, this is demonstrated in VSMCs isolated from mice [37]. Contrary to the previous findings, Zhang et al. reported that adiponectin treatment inhibits the proliferation and migration of cultured human coronary artery VSMCs [38]. They also reported that adiponectin treatment stimulates apoptosis by modulating mitofusin-2 (MFN2)-mediated Ras-Raf-Erk1/2 signaling pathway (Fig. 2) [38]. Findings from these studies suggest that adiponectin inhibits neointimal formation in pathological conditions where excessive proliferation and numbers of VSMCs are deleterious, through suppressing the proliferation and migration as well as enhancing apoptosis of VSMCs.

A study aimed to determine the role of adiponectin and its receptors in angiotensin II (Ang II)-induced vascular injury [35]; the findings from the study suggest that adiponectin treatment significantly attenuated Ang II-induced VSMCs migration and p38 phosphorylation [35]. Additionally, treatment with AdipoRon attenuated Ang II-induced hypertensive vascular hypertrophy and fibrosis [35]. Another study using rat aortic tissue showed that adiponectin pretreatment before Ang II challenge attenuated Ang II-mediated hypertrophic effect in VSMCs through the inhibition of RhoA/ROCK pathway and subsequent ROS formation (Fig. 2) [39]. Thus, it appears adiponectin works as a protective molecule in Ang II-induced hypertension following vascular remodeling.

### Adiponectin aided regulation of cardiac muscle function

**Fig. 3:**
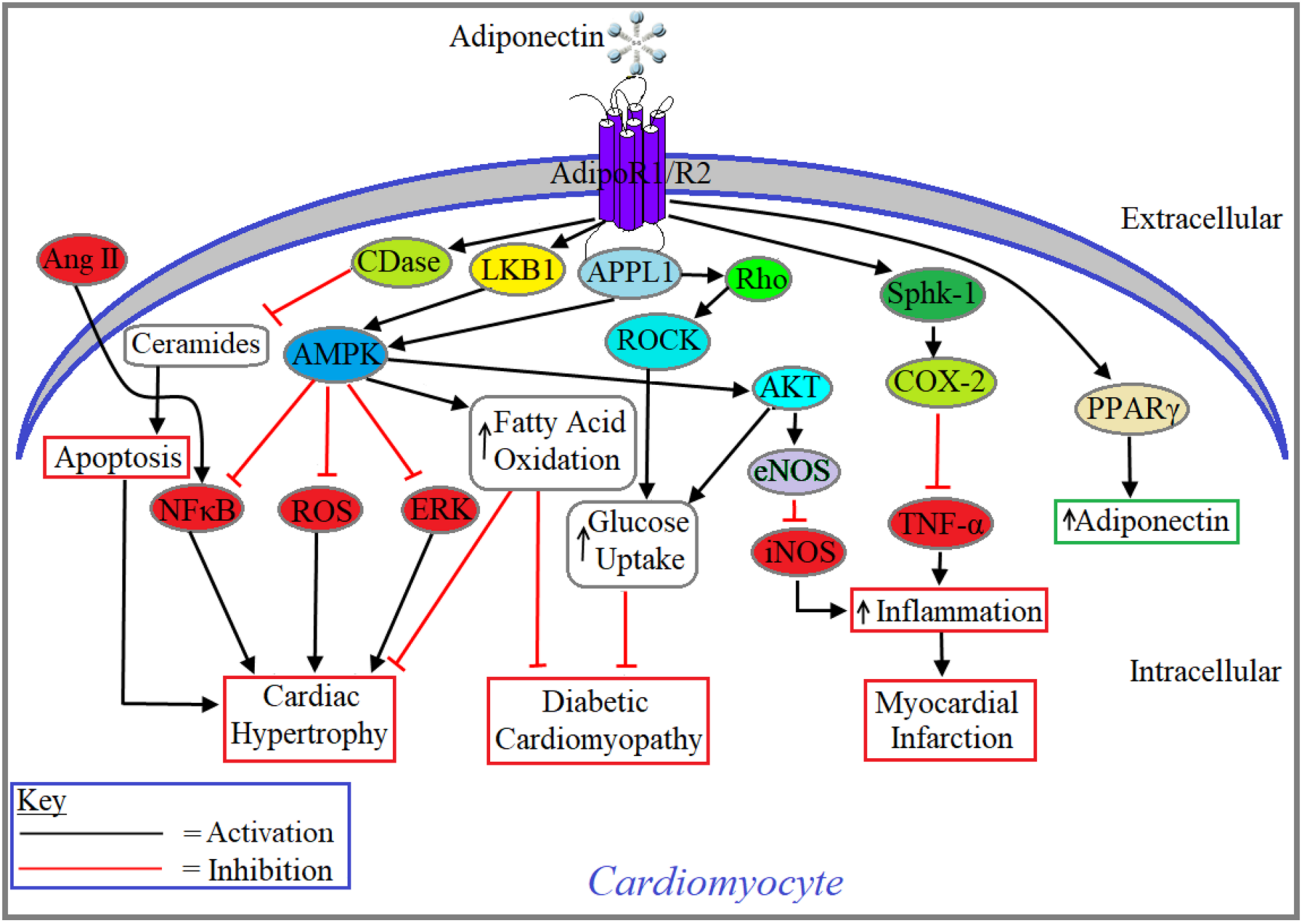
Adiponectin signaling in Cardiomyocyte. Adiponectin-induced activation of AdipoR1 or AdipoR2, triggers LKB1-mediated activation of AMPK that prevents cardiac hypertrophy by inhibiting Nuclear factor-κB (NF-ĸB), reactive oxygen species (ROS) formation and extracellular signal-regulated kinase (ERK) and by stimulating fatty acid oxidation. Adiponectin-induced AMPK also prevents inflammation-mediated myocardial infarction through the activation of endothelial nitric oxide synthase (eNOS) as well as prevents diabetic cardiomyopathy via increasing fatty acid oxidation. Adiponectin-induced activation of ceramidase (CDase) prevents apoptosis of cardiomyocytes and subsequent development of cardiac hypertrophy through the degradation of ceramides. Activated AdipoR1/AdipoR2 prevents diabetic cardiomyopathy through APPL1-Rho-ROCK-mediated elevation of glucose uptake. Adiponectin-induced activation of Sphk-1-COX-2 cascade prevents myocardial infarction through the inhibition of TNF-α-mediated inflammation. Adiponectin-induced activation of PPARγ increases the transcription of adiponectin in cardiomyocytes

Cardiomyocyte is the key cell type of the heart tissue, as it is responsible for cardiac contractility and relaxation, the primary function of the heart. Most cardiac diseases including heart failure (HF), cardiomyopathy and myocardial infarction (MI) are directly associated with impaired contractile function/death of cardiomyocytes [40]. Metabolic diseases like obesity and type 2 diabetes mellitus (T2DM) are strongly associated with heart diseases such as atherosclerosis, coronary artery disease (CAD), MI and HF [41]. Several studies in humans and rodents confirmed that obesity [42], T2DM [43], hypertension [44], atherosclerosis [45], CAD [46], MI [47] and HF [46] are associated with reduced plasma adiponectin levels and/or impaired adiponectin signaling in target tissues. Cardiomyocytes are known to secrete adiponectin in a paracrine/autocrine manner, although this level is relatively low compared with the levels produced by the adipose tissue [48, 49]. Several studies reported that adiponectin receptors are expressed in human cardiomyocytes, murine derived atrial cardiomyocytes and neonatal rat ventricular cardiomyocytes [48, 50]. Adiponectin is known to exert its cardioprotective role on cardiomyocytes through the activation of AdipoR1 and AdipoR2 [49]. Adiponectin improved contractility of cardiomyocytes isolated from *db/db* obese mice through the activation of AdipoR1/AdipoR2 [51]. Cao et al*.*, revealed that globular adiponectin attenuated Ang II-induced atrial hypertrophy through the activation of AdipoR1/APPL1 signaling pathway and subsequent decrease in NF-κB activation and translocation into the nucleus in rat atrial cardiomyocytes [52]. They also reported that activation of AdipoR1/APPL1 upregulated the expression and/or activation of downstream signaling molecules including PI3K, AMPK and NF-ĸB in rat atrial cardiomyocytes [52]. SiRNA-mediated knockdown of AdipoR1 and/or AdipoR2 attenuated C1q tumor necrosis factor-alpha-related protein 9 (CTRP9)-mediated inhibition of ROS formation and increased apoptosis of cardiomyocytes in H9C2 rat cardiomyoblast cell line [53]. Cardiomyocyte-specific PPARγ overexpressing mice showed a significant increase in adiponectin production in an autocrine manner and subsequently protect cardiomyocytes from hypertrophy that dictates PPARγ a downstream signaling molecule of adiponectin expression in cardiomyocytes [54]. Another study concluded that exenatide, a 39-amino-acid peptide and a synthetic version of exendin-4 hormone which is used as an anti-diabetic medication, prevented the apoptosis of isolated cardiomyocytes from diabetic rats via upregulating the expression of adiponectin, APPL1, p-AMPK and PPARα and downregulating the expression of NFκB [55]. Pretreatment with recombinant rat adiponectin before Ang II stimulation, promoted AMPK phosphorylation and inhibited ERK1/2 activation in neonatal rat ventricular myocytes (NRVMs) [56]. Adiponectin treatment is shown to upregulate the expression of miR-133a, a key regulator of cardiac hypertrophy through AdipoR1/AMPK activation and ERK1/2 inhibition in NRVMs [56]. Adiponectin has been shown to induce actin cytoskeleton remodeling to mobilize GLUT-4 mediated increased glucose uptake through the activation of APPL1/Rho/Rho-associated protein kinase (ROCK) signaling pathway in neonatal rat cardiomyocytes (Fig. 3) [57]. Adiponectin promotes the activation of AdipoR1/APPL1/AMPKα2 signaling and inhibits acetyl-CoA carboxylase (ACC) activity through phosphorylation and subsequently increases fatty acid oxidation in rat cardiomyocytes (Fig. 3) [58]. Additionally, adiponectin increases cluster of differentiation 36 (CD36) translocation and fatty acid uptake as well as AKT phosphorylation and insulin-induced glucose transport in primary cardiomyocytes (Fig. 3) [58]. Adiponectin-induced activation of AMPK has been shown to share its cardioprotective role with Sirtuin 1 (Sirt1). A recent study showed that adiponectin-induced activation of AMPK/Sirt1 signaling contributes to the preconditioning of myocardial ischemia–reperfusion (IR) injury in rats [59]. Adiponectin treatment is shown to induce the activation of ceramidase (CDase) and subsequently prevents apoptosis of isolated ventricular cardiomyocytes from obese mice through the degradation of ceramides [60]. Adiponectin has been shown to involve myocardial ischemic postconditioning (IPo)-mediated cardioprotection against myocardial ischemia–reperfusion (IR) injury in rodents via both AMPK-dependent nuclear and AMPK-independent mitochondrial signal transducer and activator of transcription (STAT)3 activation [61]. Adipo KO mice exhibited enhanced myocardial apoptosis, infract size, levels of nitric oxide (NO), inducible nitric oxide synthase (iNOS), superoxide, and their cytotoxic reaction products, peroxynitrite, in cardiac tissues [62]. On the other hand, adiponectin treatment markedly decreased iNOS protein expression and NO/superoxide production, blocked peroxynitrite formation and attenuated the progression of atherosclerotic lesions in carotid artery of *ApoE*^−/−^ *mice* [63]. Intraperitoneal injection of a single dose of doxorubicin, a cardiotoxic chemotherapeutic agent, was shown to significantly increase mortality and exacerbated left ventricular contractile dysfunction in adipo KO mice compared with age-matched doxorubicin-injected WT control [64] while adiponectin treatment was shown to ameliorate doxorubicin-mediated cardiotoxicity through Akt activation in NRVMs [64]. Induction of MI increased the cardiac levels of COX-2, a cardioprotective molecule, in wild type mice which was inhibited in adiponectin-deficient mice. By using primary rat cardiomyocytes, adiponectin was shown to increase COX-2 level, however to determine the signaling mechanism of whether adiponectin augments COX-2 expression through a sphingosine kinase-1 (SphK-1)-dependent pathway, the cardiac myocytes were pretreated with a SphK-1 inhibitor (SKI) followed by incubation with adiponectin or vehicle. It was found that adiponectin-mediated COX-2 upregulation was diminished by 38% by preincubation with SKI. This was again confirmed by treating the cardiomyocytes with SiRNA of SphK-1 before treating with adiponectin as the results revealed that adiponectin-induced COX-2 expression was decreased by SiRNA of SphK-1 by 43% compared to control RNA. Thus, it can be summarized that adiponectin activates sphingosine kinase-1 (SphK-1)/COX-2 signaling in cardiomyocytes and thus contributes to the cardioprotection in myocardial inf arction (Fig. 3) [65, 66].

### Adiponectin aided regulation of vascular endothelial cell function

**Fig. 4:**
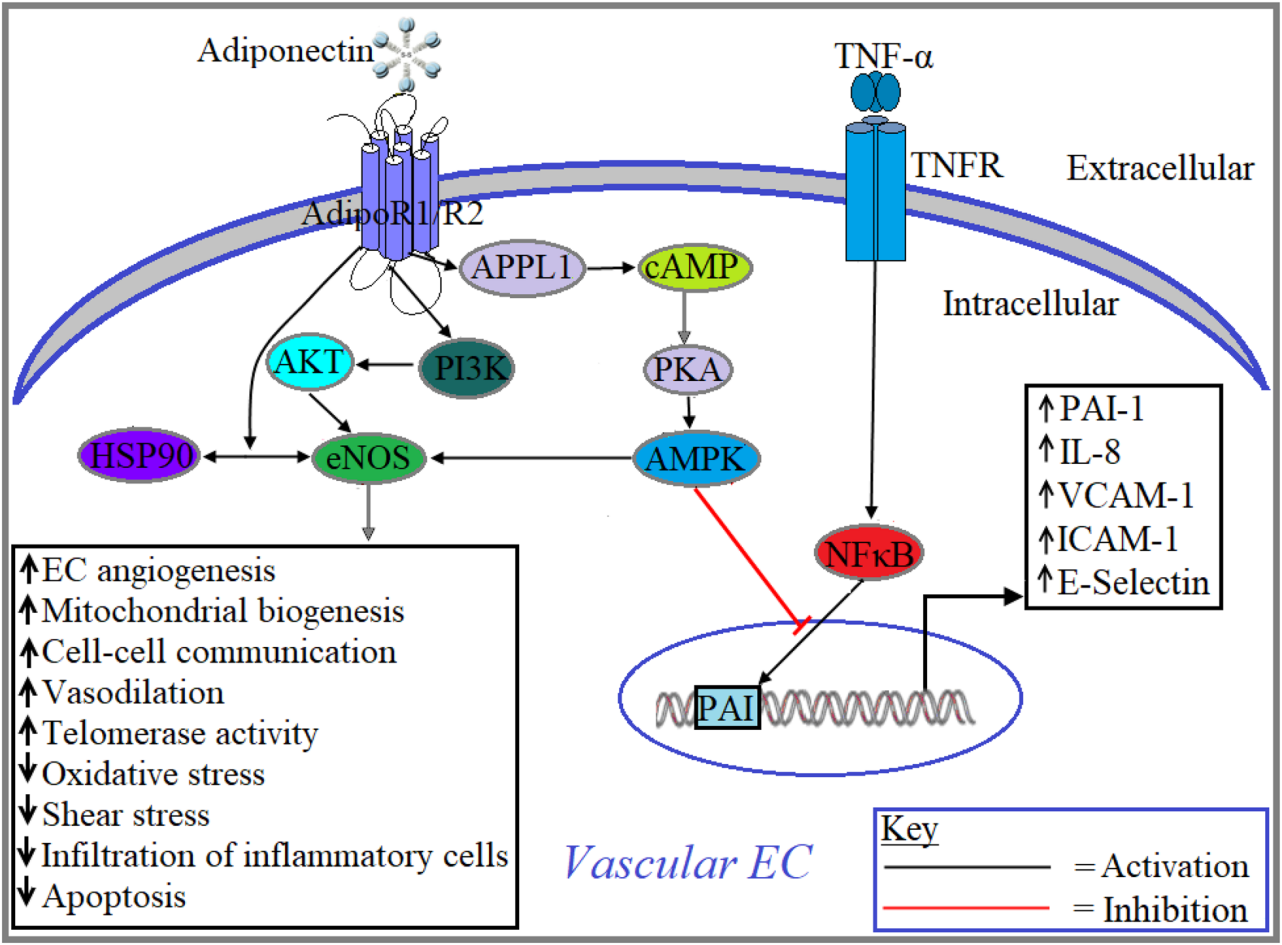
Adiponectin signaling in vascular endothelial cell. Adiponectin binds with its receptors, AdipoR1/AdipoR2, which in turn activate the downstream signaling molecules. Activation of AdipoR1 or AdipoR2 induces eNOS activation through PI3K/AKT signaling pathway and subsequently increases EC angiogenesis, mitochondrial biogenesis, cell–cell communication, vasodilation and telomerase activity, whereas it decreases oxidative stress, shear stress, infiltration of inflammatory cells and apoptosis. Adiponectin-induced activation of APPL1-cAPM-PKA-AMPK inhibits NF-ĸB-mediated expression of proinflammatory factors including plasminogen activator inhibitor-1 (PAI-1), interleukin 8 (IL-8), vascular cell adhesion molecule 1 (VCAM-1), intercellular adhesion molecule *1* (ICAM-1) and E-Selectin

The vascular endothelial cells (ECs) plays an indispensable role in the cardiovascular system as they serve as a protective barrier between blood and vessel walls, as well as secrete a plethora of bioactive substances including vasoactive substances, enzymes, growth factors, cytokines and chemokines [67]. Impaired functioning of the vascular ECs due to oxidative and shear stress, systemic hypertension, elevated levels of free fatty acids, pathogenic infection and intake of hazardous chemicals can cause severe consequences in cardiovascular physiology including coronary artery disease (CAD), MI, cardiomyopathy, HF, atherosclerosis and arteriosclerosis [68]. One of the most important factors that maintain cardiovascular homeostasis by controlling NO bioavailability is endothelial nitric oxide synthase (eNOS). The broad range of activities of eNOS in vascular ECs largely depends on posttranslational modifications, including acylation, nitrosylation, phosphorylation, acetylation, glycosylation and glutathionylation [69]. Activation of eNOS-NO signaling is associated with several cardiovascular functions including upregulation of EC angiogenesis, mitochondrial biogenesis, cell–cell communication, vasodilation and telomerase activity and downregulation of oxidative stress, shear stress and infiltration of inflammatory cells [70]. Adiponectin has been shown to induce the phosphorylation of eNOS at Ser^633^ [71] and Ser^1177^ and generation of NO in human umbilical vein endothelial cells (HUVECs) through AdipoR1/R2-APPL1/AMPK-mediated pathway (Fig. 4) [72]. Interaction between the heat shock protein (HSP) 90 and eNOS is required to produce NO in ECs which was confirmed by Amour et al. through a study where pharmacological inhibition of HSP90 activity attenuated Isoflurane-dependent NO production in human CECs (Fig. 4) [73]. Globular adiponectin treatment dose-dependently increased NO in bovine aortic endothelial cells (BAECs). NO generation was elicited by stimulating the binding of HSP90 with eNOS and subsequently activation of PI3-AKT signaling pathway to over activate eNOS. It was shown that pharmacological inhibition of HSP90 or PI3K in BAECs decreased globular adiponectin-mediated NO release. This globular adiponectin also increased endothelial cell-dependent vasorelaxation in rat aortic rings [74]. Treatment with globular adiponectin (5 μg/ml) inhibited palmitate-induced ROS generation and subsequent induction of apoptosis in HUVECs. Using pharmacological interventions for cyclic AMP (cAMP) and AMPK, this study also showed that adiponectin inhibits the generation of ROS and subsequent induction of apoptosis in HUVECs through cAMP/PKA and AMPK mediated signaling pathway (Fig. 4) [75]. Transcription factor NF-ĸB signaling plays a central role in the transcription of several proinflammatory cytokines and growth factors that are associated with the pathogenesis of several diseases including atherosclerosis [76]. Adenoviral transduction of adiponectin gene attenuated the formation of atherosclerotic plaques in the aortic arch of ApoE^−/−^ mice through the inhibition of NF-ĸB pathway (Fig. 4) [77]. Treatment with globular adiponectin has been shown to increase human microvascular ECs migration, proliferation and angiogenesis through upregulating the expression of proangiogenic factors including matrix metalloproteinase (MMP)-2, MMP-9 and VEGF. Adiponectin-induced upregulated expression of VEGF is mediated via the activation of AdipoR1, and MMP-2 and MMP-9 are mediated via the activation of both AdipoR1 and AdipoR2 [78]. Adiponectin appears to play an important role in mitochondrial biogenesis in vascular ECs as evident by a study where Adipo KO mice downregulated the expression of regulatory proteins of mitochondrial biogenesis including PGC-1α, nuclear respiratory factor 1, transcription factor A, Sirt3 and Sirt1 in pulmonary vascular ECs [79].

### Adiponectin aided regulation of hepatocyte function

**Fig. 5:**
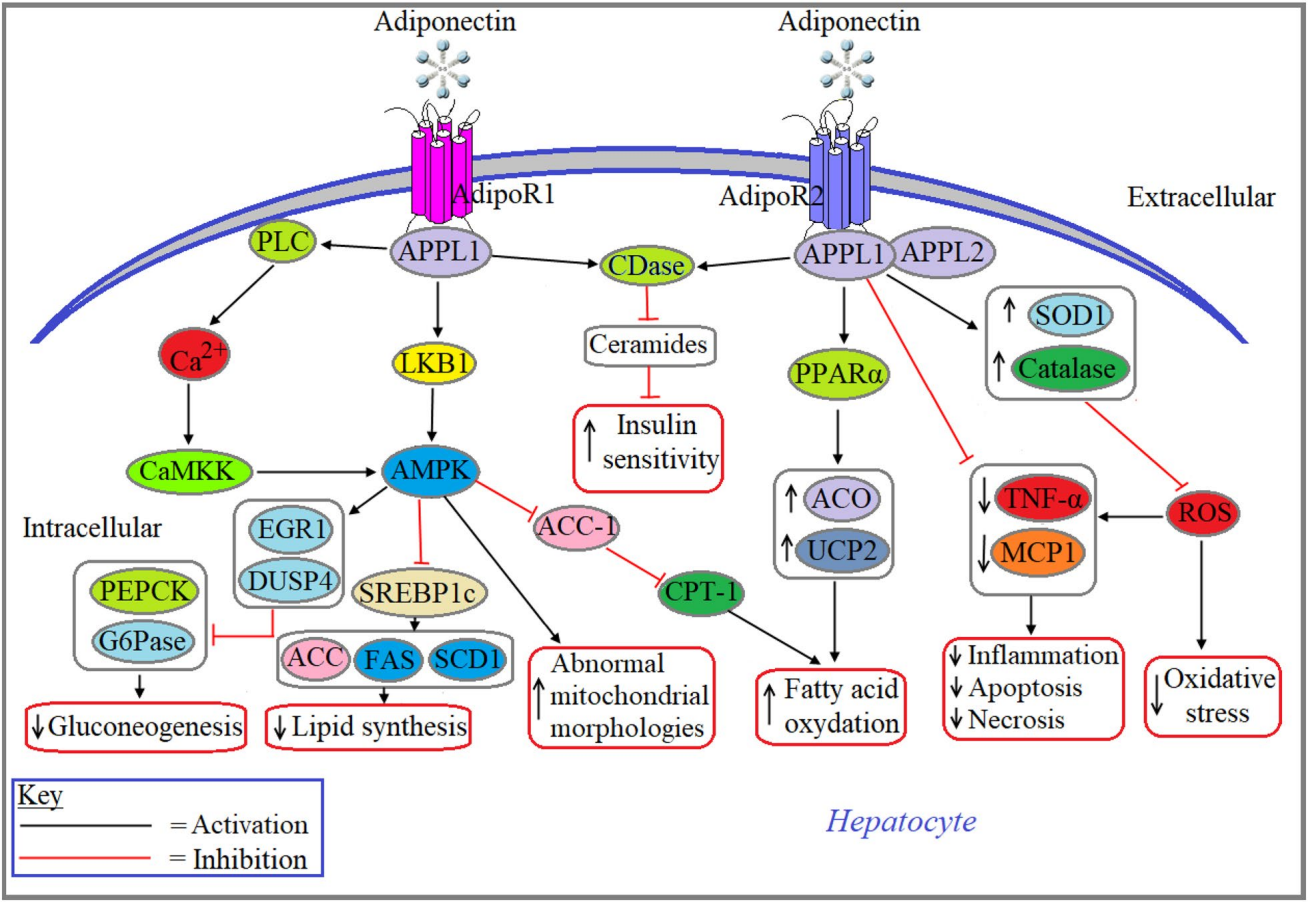
Role of adiponectin signaling in the liver. Binding of adiponectin with AdipoR1 activates AMPK via APPL1/LKB1 or APPL1/PLC/Ca^2+^/CaMKK mediated signaling pathways. Activated AMPK subsequently inhibits the activities of PEPCK and G6Pase through the activation of EGR1 and DUSP4, which leads to decreased gluconeogenesis. Activated AMPK also decreases lipid biosynthesis through inhibiting sterol regulatory element-binding transcription factor 1c (SREBP1c)-mediated transcription of acetyl-CoA carboxylase (ACC), FAS and *stearoyl-CoA desaturase* (SCD)1. Additionally, activated AMPK increases abnormality in mitochondrial morphologies and fatty acid oxidation via sequestering the inhibitory effect of ACC-1 on carnitine palmitoyltransferase (CPT)-1 activation. Independent of AMPK activation, adiponectin also increases insulin sensitivity through the activation of AdipoR1 or AdipoR2 following CDase-mediated degradation of ceramides. Upon activation of AdipoR2 with the binding of adiponectin, fatty acid oxidation increases through PPARα-induced activation of acyl-CoA oxidase (ACO) and uncoupling protein (UCP)2. Adiponectin-induced activation of AdipoR1 contributes to the activation of antioxidant enzymes, superoxide dismutase (SOD)1 and catalase which subsequently decreases oxidative stress via downregulating ROS production. Adiponectin also protects hepatocytes through the inhibition of ROS-mediated activation of proinflammatory molecules including TNF-α and MCP1

Hepatocytes, the major cell types in the liver that derived from parenchyma, contribute to the ~ 80% mass of the liver tissue and play indispensable roles in metabolism, detoxification, protein synthesis and activation of the innate immune system [80]. In hepatocytes, adiponectin mainly contributes to the regulation of glucose and fat metabolism through an insulin-sensitizing effect. The role of adiponectin in healthy liver includes a decrease in gluconeogenesis, lipid biosynthesis, inflammation, and oxidative stress, and an increase in insulin sensitivity and fatty acid oxidation [81]. Adiponectin confers its biological effects in hepatocytes through the activation of AdipoR1 and AdipoR2 [82]. A review has summarized a large number of studies to report that adiponectin-induced decrease in gluconeogenesis is regulated mainly through the activation of the liver kinase B1 (LKB1)/AMPK axis in hepatocytes [83]. Adenoviral transduction of constitutively active form of AMPK has been shown to decrease the rate of gluconeogenesis through sequestering the expression and activities of two rate-limiting gluconeogenic enzymes, phosphoenolpyruvate carboxykinase (*PEPCK*) and glucose-6-phosphatase (*G6Pase*) in primary rat hepatocytes [84, 85]. Transcriptional profiling result from another study showed that treatment with 5-aminoimidazole-4-carboxamide riboside (AICAR), an AMPK activator, upregulated the transcription of two downstream targets of AMPK, early growth response 1 (EGR1) and dual-specificity protein phosphatase 4 (DUSP4) in AML12, H4IIE, and Fao cells. This finding was again confirmed by measuring mRNA and protein levels of EGR1 and DUSP4 in rat hepatoma cell lines, H4IIE and Fao. Using a reporter gene assay and real-time qPCR, this study further demonstrated that exogenous DUSP4 inhibits the promoter activity and expression of both PEPCK and G6Pase in H4IIE cells [86]. Some reviews have summarized a large number of studies to report that adiponectin-induced activation of AMPK in hepatocytes is mediated via PLC/Ca^2+^/CaMKK signaling pathway [82, 87]. Administration of adiponectin decreased fat accumulation in the liver of +*Lepr*^*db*^/+*Lepr*^*db*^ (*db/db*) mice as well as in cultured Fao cell lines. This action of adiponectin is predominantly associated with the activation of the AdipoR1/LKB1/AMPK signaling pathway and subsequent downregulation of sterol regulatory element-binding protein 1c (SREBP1c), a master regulator of fatty acid synthesis in the liver. The involvement of AdipoR1/LKB1/AMPK signaling in adiponectin-induced downregulation of SREBP1c was further confirmed in LKB1 KO (*LKB1*^lox/lox^) mice, where the absence of LKB1 rescued the inhibitory effect of adiponectin on SREBP1c expression [88]. However, a review demonstrated that adiponectin-induced AMPK activation in hepatocytes is much lower than other cell types [89]. Adiponectin lowers the rate of gluconeogenesis in hepatocytes not only through the activation of LKB1/AMPK signaling pathway but also through other pathway/s independent of LKB1/AMPK activation [90]. Findings from this study were confirmed in mice with inducible hepatic deletion of LKB1. This study showed that deletion of LKB1 in the liver partially impaired glucose-lowering effects of adiponectin in LKB1^lox/lox^ mice. However, adiponectin-induced downregulation of gluconeogenic and lipogenic gene expression including Ppargc1a, Glucose-6-phosphatase catalytic subunit, Phosphoenolpyruvate carboxykinase 1, SREBP1c, acetyl-CoA carboxylase (ACC) alpha, ATP citrate synthase, and fatty acid synthase (FAS) in and decreased hepatic glucose production was preserved in the liver of *LKB1*^*lox/lox*^ mice [90]. Review with a large number of research findings demonstrated that adiponectin increases β-oxidation in hepatocytes through the activation of AdipoR1/LKB/AMPK signaling pathway as well as increased activation of PPARα-induced upregulation of acyl CoA oxidase (ACO) and UCP2. Adiponectin-induced inhibition of ACC-1 transcription followed by the activation of carnitine palmitoyl transferase-1 (CPT-1) facilitates fatty acid transport into the mitochondria for β-oxidation (Fig. 5) [89]. Adiponectin-induced activation of AdipoR1 or AdipoR2 prevents hepatocellular ceramide accumulation through increased activation of ceramidase (CDase) and thereby may attenuate ceramide-mediated insulin resistance and hepatocyte apoptosis (Fig. 5) [60]. This finding was confirmed with increased CDase activity and decreased ceramide accumulation in liver-specific AdipoR1 and/or AdipoR2 overexpressing mice liver [91]. Treatment with fibroblast growth factor 21 (FGF21), an important regulator of metabolism and energy homeostasis significantly decreased ceramide accumulation in hepatocytes of obese mice through increasing the expression of adiponectin, which leads to the restoration of euglycemia and reduced hyperlipidemia. The finding of this study was further confirmed when the effects of FGF21 were blunted in AdipoKO mice [92]. A study in AdipoKO mice has been shown to increase abnormal mitochondrial morphologies, decrease the oxidative activities of mitochondrial respiratory chain (MRC) complexes as well as decrease the expression of mitochondrial uncoupling protein 2 (UCP2) in the liver tissues relative to their age-matched control (Fig. 5). In the same study, the role of adiponectin in liver mitochondria was further confirmed when adenovirus-mediated replenishment of adiponectin in AdipoKO mice liver significantly decreased lipid accumulation, restored the oxidative activities of MRC complexes and reduced the formation of lipid peroxidation products compared with control mice. Although, there were no significant effects on hepatocellular mitochondrial biogenesis and ROS production in adiponectin replenished liver in AdipoKO mice [93]. Adiponectin is reported to ameliorate D-galactosamine/lipopolysaccharide (GalN/LPS)-induced liver injury in KK-Ay obese mice. A study reported that GalN/LPS-induced liver injury is associated with systemic elevation of aspartate aminotransferase (AST) and alanine aminotransferase (ALT), two important biomarkers of liver damage, increased levels of systemic and hepatocellular TNF-α, and/or showed a high degree of lethality in KK-Ay obese mice. Intraperitoneal administration of adiponectin before GalN/LPS challenge, ameliorated the GalN/LPS‐induced elevation of systemic AST and ALT levels, systemic and hepatocellular TNF-α levels as well as a reduction in lethality in KK-Ay obese mice. Additionally, adiponectin pretreatment increased the expression of PPARα mRNA in the liver of obese mice (Fig. 5) [94]. A study in 47 NAFLD patients showed systemic elevation of TNF-α, monocyte chemoattractant protein (MCP)-1 and IL-6 levels and decrease in adiponectin levels in both males and females [95]. Therefore, it can be surmised that adiponectin works as an anti-inflammatory molecule by lowering the hepatocellular proinflammatory cytokine levels. Cumulative findings from a review demonstrated that adiponectin-induced activation of antioxidant enzymes, superoxide dismutase (SOD)1 and catalase contribute to hepatoprotection through the inhibition of ROS-mediated production of proinflammatory molecules and subsequent reduction of oxidative stress (Fig. 5) [96].

### Adiponectin aided regulation of pancreatic islet function

Around 50–70% of the human pancreatic islets are β cells which are known to play a profound role in metabolism through the production, storage and secretion of insulin and amylin, two major peptides that regulate glucose homeostasis in circulation [97]. Since the cell signaling mechanism of adiponectin is associated mostly with insulin-dependent tissues, a handful of metabolic studies focus on the role of adiponectin in pancreatic islet cells. However, due to the lack of substantial findings, the role of adiponectin signaling in pancreatic α and β-cells are not completely known yet. Adiponectin exerts its biological effect on pancreatic β-cells through the activation of both AdipoR1 and AdipoR2, although AdipoR1 predominantly expressed in β-cells and globular adiponectin has a higher affinity for AdipoR1 [98–100].

**Fig. 6:**
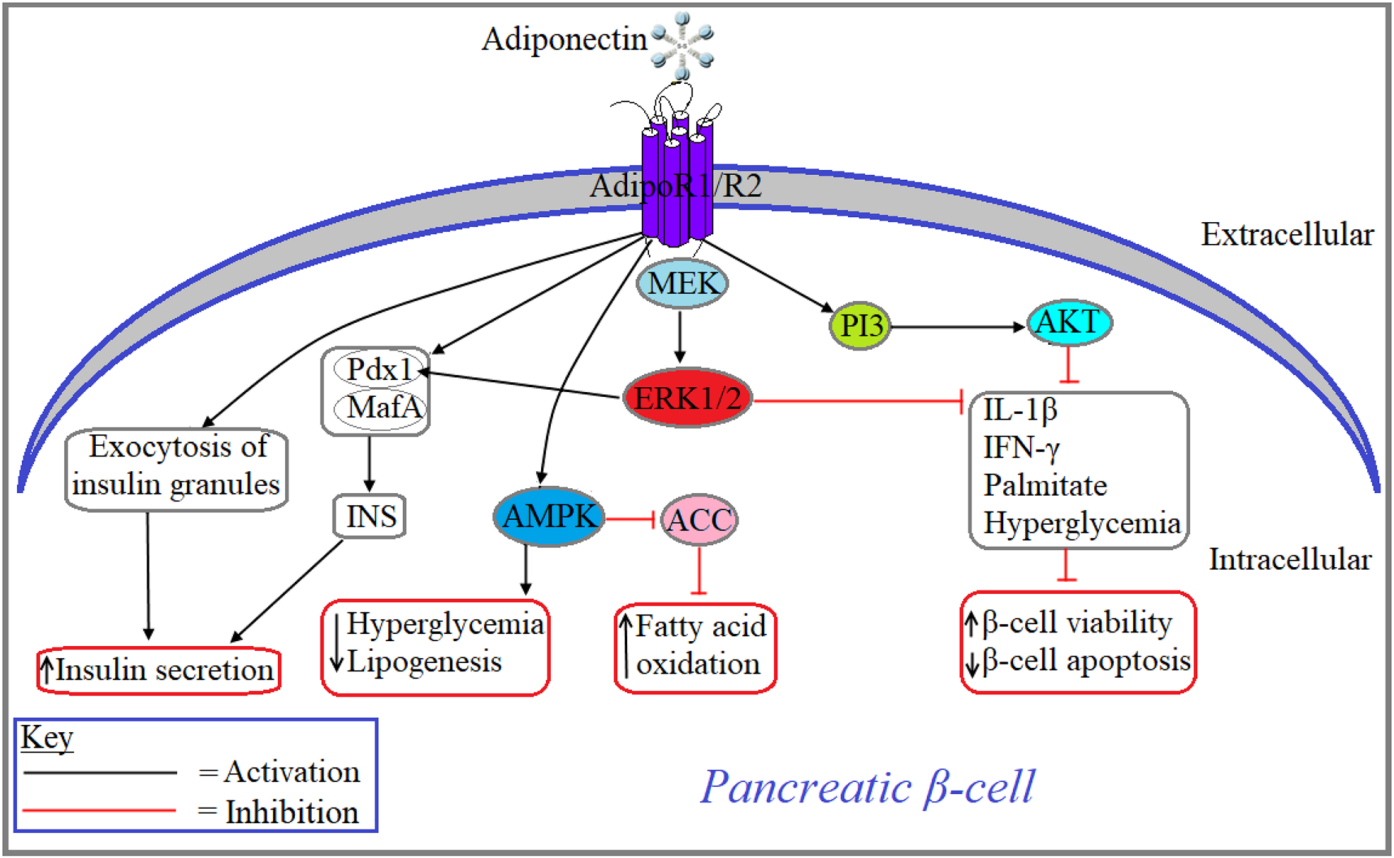
Role of adiponectin signaling in pancreatic β-cell. Adiponectin-induced activation of its receptors, AdipoR1 or AdipoR2, increases insulin secretion by increasing the transcription of insulin gene (INS) through the upregulation of transcription factors pancreatic and duodenal homeobox (Pdx)1 and MafA as well as increasing exocytosis of insulin granules. Adiponectin decreases the rate of hyperglycemia and lipogenesis through the activation of AMPK. Adiponectin-induced activation of AMPK also increases fatty acid oxidation through the inhibition of acetyl-CoA carboxylase (ACC). Adiponectin-induced activation of both MEK-ERK1/2 and PI3-AKT signaling pathways increase β-cell viability and decrease β-cell apoptosis through the inhibition of IL-1β, IFN-γ, palmitate and hyperglycemia

A study by Okamoto et al., showed that administration of full-length adiponectin facilitates insulin secretion from both isolated and intact pancreatic islet in C57BL/6 mice by enhancing exocytosis of insulin granules without affecting ATP generation, membrane potentials, cytosolic Ca^2+^ concentrations or activation status of AMPK. This study also showed that adiponectin-induced activation of AMPK inhibits glucose-stimulated lipogenesis in MIN6 beta cells (Fig. 6) [101]. Adiponectin treated isolated islets from rats for 24 h, increased the mRNA expression of PPARγ and pancreatic and duodenal homeobox (Pdx)-1, a key transcription factor that regulates early pancreas formation as well as glucose-mediated insulin secretion from β-cells (Fig. 6) [102]. Treatment with globular adiponectin for 24 h prevented apoptosis, increased β-cell viability, upregulated the expression of insulin genes (*Ins I* and *Ins II*) and secretion of insulin as through the activation of AKT and ERK in both MIN6 beta cells and isolated islets from mice. Findings from this study were further confirmed when the adiponectin-induced decrease in apoptosis, increased β-cell viability, upregulation of insulin gene expression and secretion of insulin were prevented with the use of dominant-negative Akt, PI3K inhibitor and MEK inhibitor**.** This study also showed that adiponectin treatment upregulated the expression of transcription factors Pdx1 and MafA in isolated mouse islets (Fig. 6) [100]. According to Ye and Scherer, adiponectin overexpressing mice with inducible acute β-cell ablation has been shown to regenerate pancreatic β-cells as well as mitigate local lipotoxicity by upregulating the transcription of two key transcription factors, hepatocyte nuclear factor 4α and PPARα [103]. Another study and a review by the same research group revealed that adiponectin induces fat tissues to uptake fat molecules from bloodstreams and subsequently lowers circulatory cholesterol levels in β-cell-deficient mice. This study also demonstrated that both insulin and adiponectin double deficient mice died after developing hypercholesterolemia. Findings from the studies suggest that a lack of adiponectin in insulin-deficient mice aggravates insulin-mediated lipoatrophy and hyperlipidemia to lethal levels [104, 105]. Contrary to this finding, a study showed that adiponectin (5 µg/ml) treated normal islets isolated from healthy mice had no significant effect on insulin secretion. Although, adiponectin (5 µg/ml) treatment inhibited insulin secretion in islets isolated from insulin resistant mice at 2.8 mM glucose exposure but augmented insulin secretion at 16.7 mM glucose exposure. Based on these findings, it can be concluded that dichotomous action of adiponectin on pancreatic islets is due to the lack of exact in vivo environments that mimic the underlying pathological conditions like obesity or insulin resistance [106].

### Adiponectin aided regulation of bone function

**Fig. 7:**
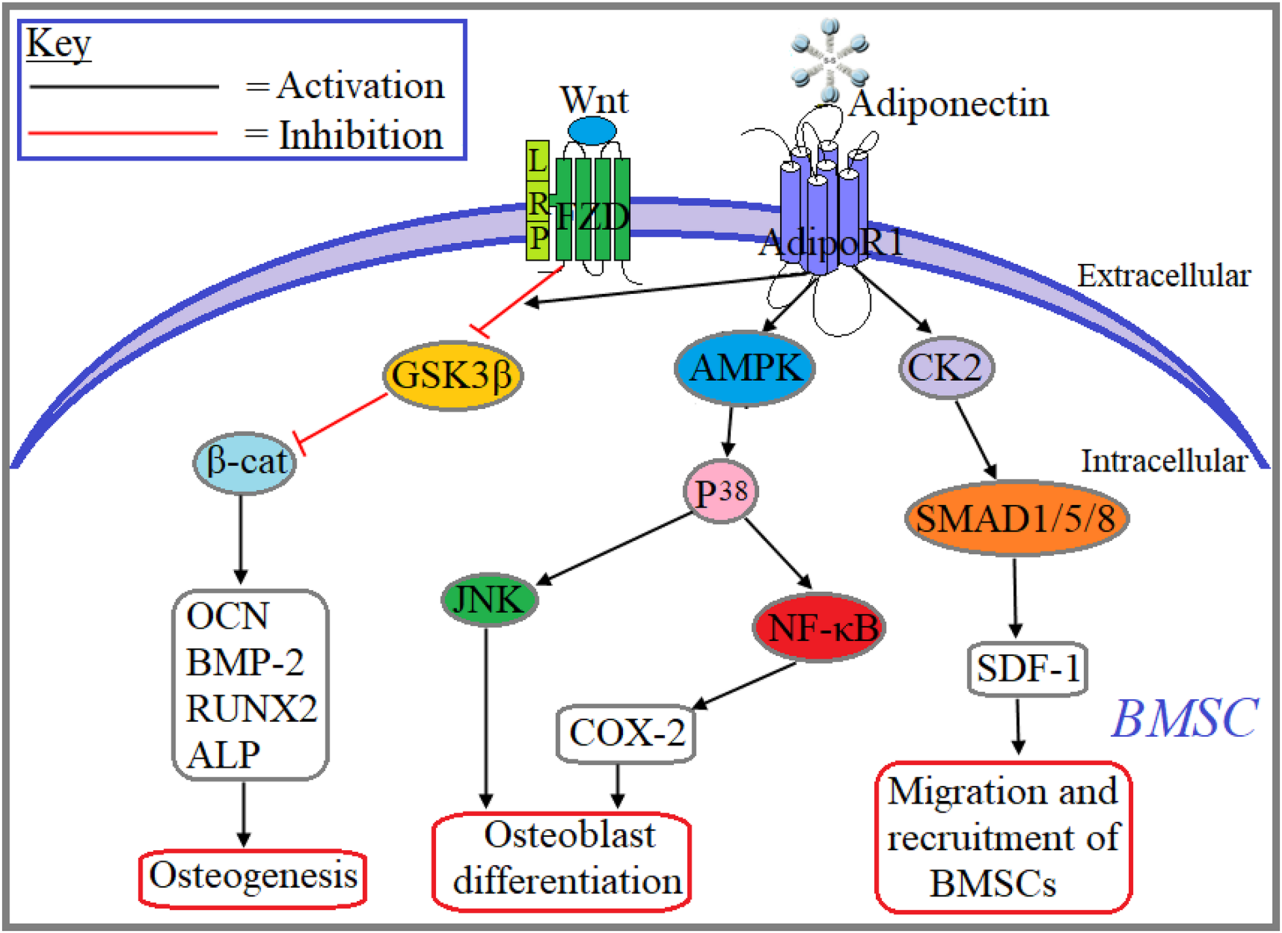
Role of adiponectin in bone marrow-derived stem cells. Adiponectin increases osteoblast differentiation through AdipoR1-AMPK-P38-NF-ĸB signaling pathway-mediated expression and activation of COX-2. Adiponectin-induced activation of P38 also contributes to osteoblast differentiation through the activation of JNK. Adiponectin signaling increases osteogenesis through the activation of Wnt/β-cat-mediated expression of osteogenic genes including OCN, BMP-2, RUNX2 and ALP. Adiponectin-induced activation of AdipoR1-CK2-SMAD1/5/8 upregulates the transcription and activation of SDF-1 and subsequently increases the migration and recruitment of BMSCs

There are three types of bone cells that contribute to the process of bone formation: osteoblasts, osteoclasts and osteocytes. Remodeling of the bone is governed by an intricate balance between osteoblast-mediated bone formation and osteoclast-mediated bone resorption. When the balance leans toward osteoblast number/activity, it causes an increase in bone mineral density (BMD) by lowering the number of osteocytes. On the other hand, when the balance leans toward osteoclast number/activity, it causes a decrease in BMD by decreasing the number of osteocytes [107]. There are limited studies that determine the role of adiponectin in bone cells. BMD data from the lumbar spine (L1–L4 together) and the hip (femoral neck) in a cohort of 92 participants showed that circulatory adiponectin levels decreased significantly in older men (60–80 years) with decreased BMD (BMI > 27 kg/m^2^) compared to older men (60–80 years) with normal BMD (BMI < 27 kg/m^2^) [108]. A study in rabbits with mandibular osteodistraction (distraction rate = 2 mm/day) showed that intermittent administration of adiponectin (2 µg) into the distraction gap promoted much greater bone formation, higher BMD and bone mineral content (BMC) compared with vehicle-treated rabbits after six weeks [109]. Administration of adiponectin overexpressing adenovirus in the jugular vein of 8-week-old C57BL/6 J male mice showed significantly larger trabecular bone volume compared with the vehicle-treated mice after 2 weeks. Additionally, treatment with recombinant adiponectin attenuated macrophage colony-stimulating factor/receptor activator of nuclear factor-κB ligand-induced differentiation of bone marrow stromal cells and human CD14-positive peripheral blood mononuclear cells (PBMCs) into osteoclasts as well as significantly reduced the resorption area of CD14-positive PBMCs relative to the control in a dose-dependent manner. Adiponectin-treated MC3T3-E1 osteoblasts significantly increased the mRNA expression of alkaline phosphatase (ALP), an important biomarker of osteoblast differentiation, and the matrix mineralization on day 12 and 18 compared with the control. Findings from this study suggest that adiponectin treatment decreases osteoclast differentiation and increases osteoblast differentiation and bone mineralization [110]. Intracerebroventricular administration of adiponectin increases serum epinephrine and norepinephrine levels as well as osteoclast numbers whereas increases trabecular bone mass as well as the number of osteoblasts in both WT and adiponectin-KO mice. Additionally, central administration of adiponectin significantly increased some neuropeptides including hypothalamic tryptophan hydroxylase 2 (TPH2) and 5-hydroxytryptamine receptor 2C (5-HTR2C). However, pharmacological stimulation of sympathetic activity with isoproterenol, a β-adrenergic agonist, attenuated adiponectin-mediated effects. These findings reveal that adiponectin increases bone mass through the suppression of sympathetic tone [111]. A study by Luo et al., showed that sustained release of adiponectin for 4 weeks in hydroxyapatite (HA) implanted ovariectomized (OVX) rabbits significantly increased peri-implant osteogenesis compared with controls and short-term adiponectin treated groups. They also showed that sustained release of adiponectin in rabbit mature osteoclast culture significantly decreased the levels of tartrate-resistant acid phosphatase and F-actin, two important histochemical markers of osteoclasts, and eventually decreased the activity of osteoclasts compared to controls as well as short-term adiponectin treatment [112]. Adenovirus-mediated transfection of adiponectin in rat bone mesenchymal stem cells (BMSCs) significantly increased the expression of AdipoR1 and AdipoR2. Treatment with adiponectin for 12 h significantly increased the mRNA expression and protein levels of β-catenin and cyclinD1, two important factors in Wnt/β-catenin signaling in cultured BMSCs. Adiponectin treatment for 3 and 7 days significantly increased the mRNA expression of some osteogenic genes including ALP, osteocalcin (OCN), bone morphogenetic protein-2 (BMP-2) and runt-related transcription factor-2 (RUNX2) in cultured BMSCs. Additionally, endogenous expression of adiponectin via intramuscular adenoviral delivery significantly increased the expression/activation of OCN, BMP-2, RUNX2 and ALP in isolated rat BMSCs (Fig. 7). Findings from this study suggest that adiponectin inhibits osteoclast-mediated bone resorption, increases osteoblast-mediated bone formation, and subsequently improves bone density [113]. A study by Huang et al., showed that adiponectin treatment increased the mRNA expression and protein levels of BMP-2, an important factor for bone remodeling, in both cultured human osteoblast-like cell lines including hFOB and murine primary osteoblastic cells. They confirmed the involvement of AdipoR1 in adiponectin-mediated BMP-2 expression as adiponectin treated osteoblast significantly increased the mRNA expression of AdipoR1. This finding was further confirmed when AdipoR1 siRNA treatment abolished adiponectin-induced expression of BMP-2 in the hFOB cell line. They also confirmed the involvement of AMPK, p38 and NF‐κB in adiponectin-induced BMP-2 expression when treatment with specific pharmacological inhibitors and siRNA blunted the effect of adiponectin in BMP-2 expression in osteoblasts. Findings from this study suggest that adiponectin-induced upregulation of BMP-2 in osteoblasts is carried out through the activation of AdipoR1/AMPK/p38/NF-ĸB signaling pathway (Fig. 7) [114]. Another study showed that adiponectin-treated mesenchymal stem cell (MSC) line C3H10T1/2 and mouse PBMCs significantly increased the mRNA expression of ALP, OCN, OPN and type 1 collagen. This study also showed that adiponectin treatment for 3 h significantly increased the mRNA expression and protein levels of cyclooxygenase (COX)-2, an important marker for osteoblast differentiation, as well as increased the levels of prostaglandin E_2_ (PGE2), the product of COX2, in C3H10T1/2 and mouse primary bone marrow cells. Adiponectin treated C3H10T1/2 cells also increased the mRNA expression of c‐Jun, an important transcriptional activator of COX2 promoter, and ChIP assay deciphered the involvement of c-Jun in COX2 promoter activity. Additionally, adiponectin increased the phosphorylation of c-Jun through the activation of p38 MAPK as confirmed by kinase assay when adiponectin treatment augmented the phosphorylation of both p38 MAPK and c-Jun. This finding was further confirmed by decreased phosphorylation of c-Jun when C3H10T1/2 cells were co-treated with p38 MAPK inhibitor. During osteogenesis, adiponectin exerts its role in MSC through the activation of AdipoR1, which was confirmed by decreased phosphorylation of p38 MAPK and COX2 expression when C3H10T1/2 cells were treated with AdipoR1 siRNA. Adiponectin-induced upregulation of BMP-2 is also mediated through the activation of COX2 which was confirmed by decreased expression of BMP-2 when C3H10T1/2 cells were co‐treated with COX2 inhibitor. Findings from this study revealed that adiponectin augmented osteoblast differentiation through AdipoR1/p38 MAPK/c-Jun-mediated transcription of cyclooxygenase (COX)-2 and subsequent upregulation of BMP-2 in mesenchymal progenitor cell (Fig. 7) [115]. To study the role of adiponectin in osteoblast retention ability within the bone marrow or at the injured bone site, Yu et al., showed that primary BMSCs isolated from 6‐week‐old APN^−/−^ mice decreased the expression of stromal cell-derived factor (SDF)-1 that mediates the retention and mobilization of stem cells. They also found that adiponectin (10 µg/ml) treated BMSCs isolated from WT mice increased MMP9 mRNA expression around tenfold and subsequently increased their migration. Additionally, the study revealed that systemic administration of adiponectin is shown to improve calvarial bone defects in DIO mice after 14 days. Also, they confirmed the involvement of SMAD1/5/8 activation in adiponectin-induced SDF-1 upregulation by repressing the expression of SDF-1 when MC3T3‐E1 cells were co-treated with adiponectin and SMAD1/5/8 inhibitor. Furthermore, they deciphered that adiponectin‐dependent Smad1/5/8 phosphorylation depends on the interaction between AdipoR1 and casein kinase II (CK2). Findings from this study suggest that adiponectin increases retention and mobilization of BMSCs and subsequently increases bone regeneration through AdipoR1/CK2/SMAD1/5/8-mediated upregulation of SDF-1 (Fig. 7) [116].

## Adiponectin as a biomarker and a therapeutic target

Studies in both humans and animals show that circulatory adiponectin levels are negatively correlated with various pathological conditions including CVDs, T2DM, obesity, insulin resistance, NAFLD, atherosclerosis, cardiomyopathy and HF [117]. Decreased levels of circulatory adiponectin are associated with a greater risk of morbidity and mortality in patients with CVDs, whereas increased circulatory adiponectin levels are cardioprotective [117]. A cohort study showed that elevated levels of circulatory adiponectin are associated with a reduced risk of developing T2DM and subsequent reduction in cardiovascular risk [118]. Consistent with this finding, the cardioprotective role of adiponectin is also revealed by other studies where chronic low levels of circulatory adiponectin is associated with the development of MI and subsequent development of cardiovascular complications in both men and women [119]. Contrary to these findings, other studies reported that hyperadiponectinemia is associated with increased risk of HF and acute coronary syndrome in both men and women [120] as well as increase the severity of post-stroke outcomes [121]. NAFLD patients with advanced hepatic fibrosis have shown a reduction in circulatory adiponectin levels [122, 123]. Additionally, decreased serum adiponectin levels are inversely correlated with insulin resistance in patients with NAFLD [124]. Contrary to these findings, other studies in humans showed that increased serum adiponectin levels are positively correlated with cirrhosis [125]. Elevated serum adiponectin levels are considered as a biomarker of renal dysfunction [126], impaired renal metabolic environment [127] and end-stage renal disease [128] in patients with CKD.

It is well-researched that pharmacological elevation of circulatory adiponectin levels can ameliorate obesity-related MetS. Studies in both animals and humans showed that using PPARγ agonist including thiazolidinediones (TZD), a diabetic medication, can increase the levels of circulatory adiponectin [129, 130]. Medicinal plant-derived natural compounds such as astragaloside II and isoastragaloside I were shown to alleviate hyperglycemia as well as improve glucose tolerance and insulin sensitivity through increasing the production of adiponectin in obese mice [131]. Extract from Zataria multiflora, a plant of the Lamiaceae family, is shown to increase PPARγ-mediated circulatory adiponectin levels and subsequent improvement of insulin sensitivity and reduced glucose levels in insulin-resistant rats [132]. A dietary supplement such as vitamin E increases plasma adiponectin mRNA expression and protein levels in mice through the activation of PPARγ signaling [133]. Additionally, adiponectin mRNA expression and protein secretion were elevated in 3T3-L1 cells after vitamin E treatment [133]. Treatment with Wy-14643, a PPARα agonist is shown to increase the mRNA expression of AdipoR1 and AdipoR2 in epididymal white adipose tissue (EWAT) of obese diabetic mice [134]. Treatment with Rosiglitazone increased the mRNA expression of AdipoR1 and AdipoR2 in isolated ventricular myocytes from rats [50]. Expression of AdipoR1 and AdipoR2 mRNAs also increased in Pioglitazone treated human skeletal muscle [135]. Administration of telmisartan, an angiotensin receptor blocker, is shown to upregulate the expression of myocardial adiponectin and AdipoR2 as well as abdominal aortic AdipoR1 in diabetic rats [136]. The administration of metformin, a well-established diabetic drug, is associated with the upregulation of AdipoR1 and AdipoR2 in skeletal muscle and AdipoR1 in rat EWAT [137].

## Concluding remarks and future directions

Nowadays the molecular insights of adipocyte biology and associated adipokines are gaining more attention towards the discovery of novel therapeutics for treating MetS and associated complications. Among all the adipokines characterized so far, adiponectin got more attention due to its pleiotropic role over the other adipokines. A vast majority of adiponectin research in humans and animals studied only the correlation between different diseased states and circulatory adiponectin levels. However, the tissue-specific expression, as well as the cell signaling mechanism of adiponectin, are not well characterized so far. More research needs to be carried out in animal and human models to establish tissue-specific precise mechanisms of adiponectin in different diseased conditions.

**Fig. 8:**
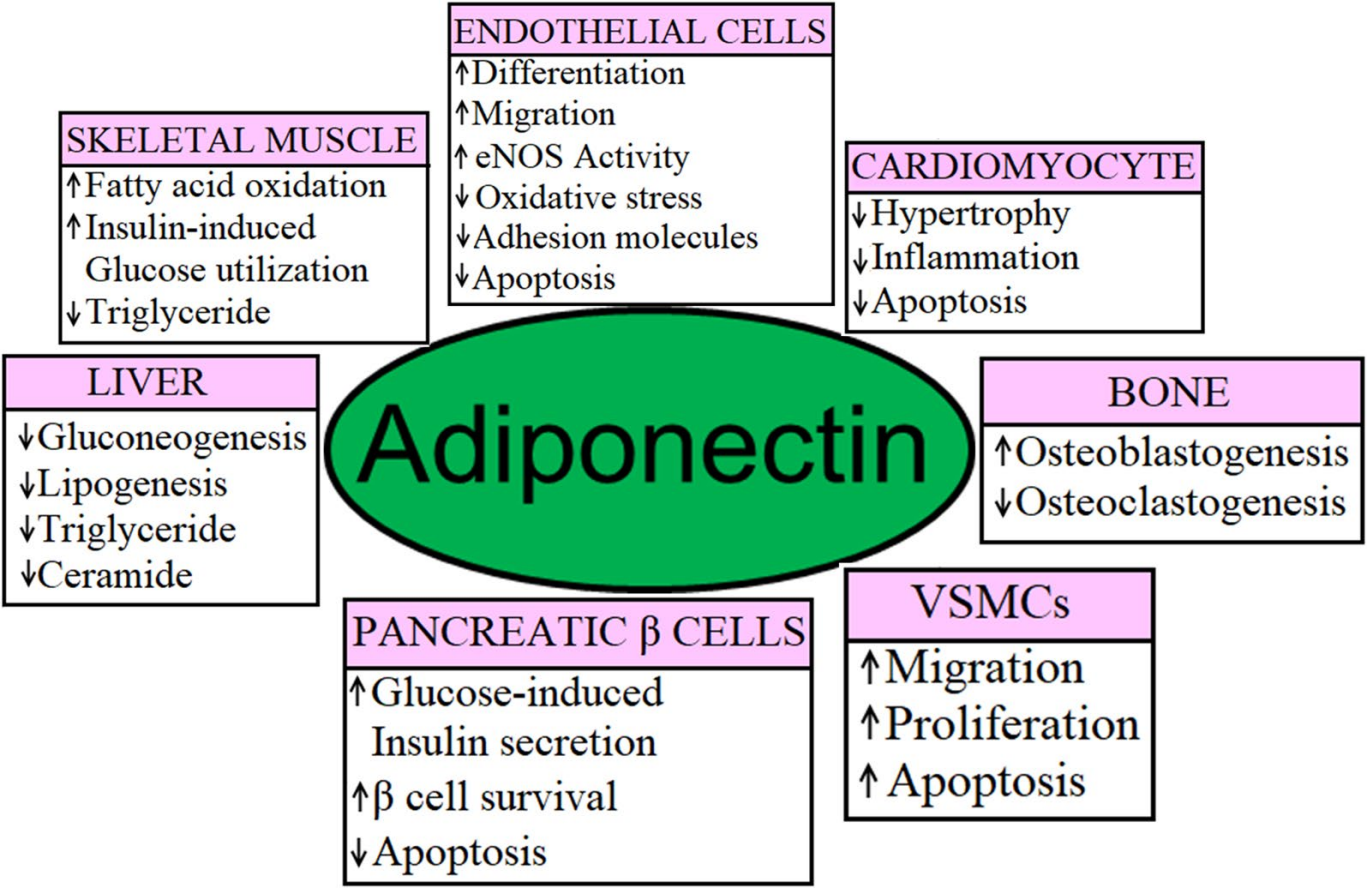
Tissue-specific role of adiponectin

Circulatory adiponectin plays important roles as a biomarker in MetS and associated CVDs and has cardioprotective, insulin-sensitizing and direct beneficial metabolic effects. Therefore, pharmacological or genetic interventions can be used to increase the levels of systemic adiponectin in MetS and associated CVDs. Additionally, adiponectin-induced activation of AdipoR1/AdipoR2 is associated with multiple signaling pathways in a wide range of cell types including skeletal muscle cells, cardiomyocytes, vascular ECs, VSMCs, hepatocytes, pancreatic β-cells and BMSCs (Fig. 8). Discovering potential therapeutics that can modulate the tissue-specific expression/activation of adiponectin and its receptors AdipoR1/AdipoR2 can pave the way to treat tissue-specific effects of MetS.

Most of the pharmacological approaches studied so far used to enhance the levels of circulatory adiponectin as well as PPARα/PPARγ-mediated increase in AdipoR1/AdipoR2 expression in humans and rodents. However, there are several downstream targets of adiponectin signaling including AMPK, mTOR, PGC1α, RhoA and SMADs in different tissue types (Figs. 1, 2, 3, 4, 5, 6, and 7) that can be used as therapeutic targets to modulate the tissue-specific activities of adiponectin in different pathological conditions. Caloric restriction and exercise training have already been proven as effective lifestyle interventions to elevate systemic adiponectin levels in patients with MetS. Therefore, simultaneous use of pharmacological and lifestyle interventions can potentiate the elevation of circulatory adiponectin levels in MetS.

## Data Availability

Not applicable.
